# Which learning techniques supported by cognitive research do students use at secondary school? Prevalence and associations with students’ beliefs and achievement

**DOI:** 10.1186/s41235-024-00567-5

**Published:** 2024-07-06

**Authors:** Héctor Ruiz-Martín, Fernando Blanco, Marta Ferrero

**Affiliations:** 1 International Science Teaching Foundation, Brighton, UK; 2 Universidad Autónoma de Madrid, Madrid, Spain; 3 Universidad de Granada, Granada, Spain

**Keywords:** Study techniques, Learning strategies, Achievement, Self-efficacy, Goal orientations, Mindsets, Exam anxiety

## Abstract

Research in cognitive science has highlighted the effectiveness of several learning techniques, and a number of studies have analyzed their prevalence among university students and their relationship with academic achievement. In this study, we surveyed a large, heterogeneous sample of secondary school students to reveal how often they use research-supported techniques in comparison with other frequent techniques, and we analyzed the association between their study strategies and school achievement. We also assessed the associations between study techniques and several students’ beliefs and attitudes toward learning (self-efficacy, goal orientation, control beliefs, growth mindset, and examination anxiety). Results showed that, except for distributed practice, only those techniques that are supported by previous research yielded an association with achievement, and they exhibited higher associations with self-efficacy, growth mindset, control beliefs, and learning goal orientation than non-supported techniques.

**Supplementary Information:**

The online version contains supplementary material available at 10.1186/s41235-024-00567-5.

## Significance statement

Revealing the most effective learning techniques is crucial for fostering academic success and student well-being. In recent years, a growing body of both basic and applied research on actions and circumstances that promote durable and transferable learning has identified promising learning strategies that may help students achieve their academic goals. It has also cautioned against some widely popular techniques that lack support from research as effective ways to invest study time. In this regard, previous research has already analyzed the study strategies commonly employed by college students and their relation to academic achievement. The present study expands on this research by examining a large and diverse sample of secondary school students, shedding light on the techniques they commonly use and how these are related to their school grades. Do the most successful students employ learning strategies supported by cognitive research? The study also analyzes how several attitudes and beliefs about learning are associated with the use of these specific study strategies. Do students who utilize effective study strategies feel more confident and experience less exam anxiety? Are they motivated by a desire for deep learning or merely good grades? The results indicate that only those techniques supported by cognitive research, specifically those based on elaborative study and retrieval practice (self-assessment), show an association with achievement. Furthermore, these techniques exhibit higher associations with positive attitudes toward learning than non-supported techniques. In sum, these findings provide valuable insights that can inform educational practices, ultimately contributing to a more supportive learning environment for students from diverse backgrounds.

## Introduction

So far this century, cognitive psychology has invigorated the interest in applied research on learning techniques. This interest has been driven by the growing body of research on the actions and circumstances that promote durable and transferable learning (Carpenter et al., 2022; Weinstein et al., 2018a, 2018b). Although study skills and learning strategies have been an important topic in education for more than 70 years (McCombs, 2017), this line of work in cognitive science has opened a new perspective in the study of the effectiveness of learning strategies that students engage in and the possibility to transfer these findings to the classrooms to improve education (Dunlosky et al., 2013; Putnam et al., 2016). For instance, research on the effects of retrieval practice (Karpicke & Roediger, 2008; Roediger et al., 2011) and its combination with spaced practice (Carpenter et al., 2022; Latimier et al., 2021), among others, have encouraged cognitive psychologists to turn their attention to applied issues in the field of learning techniques (e.g., Agarwal et al., 2021; Bjork & Bjork, 2011; Dunlosky & Rawson 2015; Putnam et al., 2016). The growing number of scientific articles dealing with these topics in the last decade attests to this trend. By way of example, a search for the term "retrieval practice" on Google Scholar yields 13,700 results, with 11,200 published after 2010. This increasing enthusiasm for uncovering the effectiveness of learning techniques may be associated with the blooming interest of the educational community in the contributions that cognitive science (and other scientific disciplines) can bring to education: the so-called “evidence-informed education” (Tokuhama-Espinosa, 2015).

The interest in transferring research on learning strategies to practice has been reflected in several reviews (e.g., Carpenter et al., 2022; Dunlosky et al., 2013), books (e.g., Brown et al., 2014; Firth, 2018; Ruiz-Martin, 2020; Weinstein et al., 2018a, 2018b), and even websites (*Unleash the Science of Learning – Retrieval Practice*, n.d.; *The Learning Scientists*, n.d.) authored by cognitive researchers. Most of these works agree on emphasizing techniques that involve retrieval practice, distributed practice, and elaborative study. One of the most cited works to this regard is Dunlosky et al.’s monograph (2013), which provides "promising directions from cognitive and educational psychology" by discussing in detail 10 learning techniques, and offering recommendations about their relative utility. The review concludes that only practice testing (retrieval practice) and distributed practice deserve a high utility rating because they turn out to be beneficial for students of different ages and abilities and have been shown to improve students’ performance in a variety of learning tasks and educational contexts. Elaborative techniques received moderate utility ratings because evidence of their efficacy is limited. Furthermore, five techniques received a low utility assessment, especially summarization, highlighting, and rereading, because they do not consistently boost students’ performance. They were nonetheless included in the review because students frequently report using them.

Once the most promising learning techniques according to cognitive psychology have been identified, the next relevant topic for research has been finding out how students put those specific techniques to use and relating this use to academic achievement. So far, the greatest bulk of this research has been conducted with university students (e.g., Blasiman et al., 2016; Bartoszewski & Gurung, 2015; Hartwig & Dunlosky, 2012; Karpicke et al., 2009; Kornell & Bjork, 2007; Yan & Wang, 2021). Results indicate that undergraduate students usually rely on relatively ineffective strategies and mass their study sessions a day or two before an examination. When comparing students’ strategies with their achievement (e.g., GPA), a positive relationship between scientifically supported strategies and academic outcomes usually emerges, although certain poor strategies, such as rereading, also show remarkable associations. For example, Hartwig and Dunlosky (2012) surveyed 324 psychology undergraduates regarding their study strategies and analyzed their association with GPA. The survey focused on techniques routinely employed by students according to previous research, such as rereading, recopying notes, and highlighting (Karpicke et al., 2009; Kornell & Bjork, 2007; Taraban et al., 1999), and those recommended by cognitive research, especially retrieval practice and spacing (Carpenter et al., 2022). While retrieval practice (self-testing) and rereading were strongly linked with high achievement, mass practice (as opposed to spacing out learning sessions) was more commonly associated with low performance.

Additionally, Bartoszewski and Gurung (2015) assessed the extent to which college students used the learning techniques described in Dunlosky et al.’s review (2013) and tested how they correlated with examination scores. In this case, students reported high use of learning techniques involving retrieval practice, and the use of many techniques were correlated with each other. However, only elaborative interrogation predicted examination scores in a multiple regression analysis.

The relationship between learning strategies and students’ beliefs toward learning has also been the object of research, although most of it has been focused on the difference between deep versus surface learning strategies (Haggis, 2003). As stated by Yan and Wang (2021), “the existing literature linking implicit beliefs and value to study strategies has not been updated to include the more recent findings about effective study strategies from cognitive psychology.” This is especially relevant for retrieval practice. Nevertheless, previous research has explored how the type of goals students adopt (i.e., performance goals vs. learning goals) influences their study behavior, including the learning strategies they choose, the effort they put forth on the task, and their persistence and time spent on task (Harackiewicz et al., 2002). Additionally, research has analyzed the associations between study behaviors and beliefs related to the nature of intelligence or academic ability (Dweck, 2000), self-efficacy (Pajares, 1996), and perceptions of control (Zimmerman & Schunk, 2006). Regarding the relationship between students’ examination anxiety and the study strategies that they use, there are already some studies that examine the case of retrieval practice. However, these studies are limited and have only been conducted with college students (Agarwal et al., 2014).

### Purpose of the study

The present work aims to extend previous research with college students—with its virtues and limitations—to secondary education students (grades 7th to 10th) in a large and heterogeneous sample. Our goal is to find out how pre-university students—many of whom will not pursue a university degree after their compulsory education—face academic challenges that require self-regulation skills, and how the techniques they use are associated with their school achievement and with their attitudes and beliefs toward learning. The importance of this research is related to the concept of “self-regulated learning”—the idea that learning outcomes are influenced by the degree in which students intentionally and proactively engage in their own learning process by setting their goals, planning and choosing their study strategies to achieve those goals, monitoring their progress, evaluating the effectiveness of their strategies, and adapting their approaches as a function of their results (Zimmerman & Schunk, 2011). For decades, the self-regulated learning framework has been a significant focus of research, leading to numerous theoretical models rooted in various perspectives (Panadero, 2017). However, they typically agree on the vital importance of learners' initiative and actions in achieving learning objectives. And although learning strategies (i.e., what students do to learn) are just one element of this wide framework, they represent a practical and relevant approach to interventions aimed at fostering the development of self-regulation and, eventually, improving academic outcomes. Considering the relevance that the concept of “learning to learn” has been given by educational administrations worldwide as a means to promote self-regulated learning (Crick et al., 2014), it is imperative to analyze whether the differences among students regarding their study behaviors are associated with their achievement not only at the college level, but also during compulsory education. In addition, since in the last years cognitive psychology has provided new insights about which learning strategies are more helpful, research analyzing the associations between these strategies, students’ beliefs, and academic outcomes becomes even more timely.

To that end, we collected the grades of a large sample of secondary school students and administered two surveys to them. The first survey included questions about students' strategies, while the second survey assessed some of their attitudes and beliefs about learning and their reported level of anxiety when facing examinations.

Our research questions were: (1) How frequently do secondary school students use techniques supported by cognitive research when they study? (2) Are these study strategies related to achievement as measured by grades? (3) Are these strategies associated with students’ beliefs and attitudes toward learning and their reported level of anxiety when facing examinations? In addition, we investigated whether the participants in this study perceived that they had been taught how to learn, as well as the differences among them concerning learning strategies, beliefs, and outcomes.

#### Surveyed study techniques

Since our study aimed to reveal secondary school students’ study behaviors supported by cognitive research and subsequently compare them to those not supported, we created surveys tailored specifically for this study. Interestingly, one of the most robustly supported learning strategies by cognitive psychology, retrieval practice (Carpenter et al., 2022; Roediger & Butler, 2011), is often overlooked in popular questionnaires on learning strategies, such as the Motivated Strategies for Learning Questionnaire, MSLQ (Pintrich et al., 1991), particularly in terms of explicit self-testing as a strategy. Unlike many studies previously conducted with college students, we organized our learning strategies survey in five categories, grouping items according to the underlying principle of learning derived from cognitive research.

Firstly, we wanted to uncover whether students spaced out their study sessions (i.e., distributing repeated practice opportunities spread over time) or, on the contrary, they massed their sessions. Indeed, the effect of distributing practice in making learning more durable (i.e., the spacing effect) is one of the most robust findings in cognitive research on memory and learning. More than a century after its first formal description (Ebbinghaus, 1885), a great number of studies have reported its benefits for learning across domains and levels of education (Carpenter 2012; Carpenter 2022; Kang, 2016). Therefore, some of the items in the survey were designed to assess how students distribute their learning over time.

Secondly, we were interested in measuring the study behaviors that should be more effective to promote long-term learning because they involve an active processing of information in terms of meaning or elaboration. Elaboration is thought to promote the organization, connection, and integration of new information with what was already known, which would make it easier to recall that new information in the future (Fiorella & Mayer, 2016). In practical terms, elaboration is a broad idea that encompasses any technique that is aimed at understanding what is learnt (as opposed to rote learning) or that actively looks for relationships between what is known and what is intended to be learned. Many cognitive scientists believe that elaboration is one of the most fundamental principles to enhance learning since long-term memory is thought to work by making connections based on meaning. By way of example, Anderson (1983, p. 285) points out that “one of the most potent manipulations that can be performed in terms of increasing a subject’s memory for material is to have the subject elaborate on the to-be-remembered material.” In fact, techniques that imply elaboration have already been explicitly included and categorized as such in widely used tests that assess students’ studying habits (Weinstein & Mayer, 1983). Accordingly, we created a category that included items for techniques that may involve a significant degree of elaboration, such as summarizing, creating conceptual maps, or generating new examples.

In spite of the theoretical superiority of elaborative study techniques, previous surveys of college students have shown that they frequently rely on techniques that do not usually involve a high level of elaboration (Blasiman et al., 2016; Bartoszewski & Gurung, 2015; Hartwig & Dunlosky, 2012; Karpicke et al., 2009; Kornell & Bjork, 2007). This is the case, for example, of rereading, highlighting, copying notes or the textbook, or trying to memorize content with a minimum level of understanding (rote learning). Therefore, we were also interested in estimating the prevalence of these behaviors at the secondary school level. Since all these study behaviors could be regarded as “low elaboration” (also referred to as “passive strategies” in other works, e.g., Yan & Wang, 2021; Zepeda & Nokes-Malach, 2021), we merged them into another category, hypothesizing that its score would not be related to achievement.

Of course, we were also particularly interested in identifying study behaviors that involved retrieval practice, that is, the act of retrieving from memory what was learned in order to explain it or use it (Roediger & Butler, 2011). Over the last few years, retrieval practice has received a lot of attention from research as a promising strategy with multiple benefits for learning (Carpenter et al., 2022; Putnam et al., 2016; Roediger & Butler, 2011; Roediger et al., 2011). The specific techniques that involve retrieval practice include self-testing, reciting (as in the "Read, Recite, Review" strategy suggested by Putnam et al., 2016), teaching peers, etc. In this respect, we created 7 items which described different behaviors associated with retrieval. Since some of these behaviors could also be regarded as elaborative techniques (e.g., teaching peers), some items in our survey were included in both categories. Likewise, an item asking about the technique of reciting a text by heart, which implies retrieval practice but with a low level of elaboration, was included in the categories of “retrieval” and “low elaboration.”

Finally, we also aimed to analyze study behaviors that are coherent with cognitive load theory (Sweller, 2011). In the last decade, cognitive load theory has become one of the most prominent theories informing education (Lovell, 2020), so we found it convenient to include some related items. Specifically, we examined the most evident and easy-to-reveal study behaviors related to the theory, that is, behaviors addressed to manage extraneous cognitive load during study in order to focus cognitive resources on the study task at hand. Many other learning techniques would be aligned with the multiple consequences for learning derived from cognitive load theory (Kirschner, 2002), but we limited our analysis to those that can be surveyed more easily. Accordingly, the items included in this category asked students, for example, whether they used to listen to music while studying or whether they used to study in a quiet environment. Therefore, this scale is related to what previous research about learning strategies in the education literature has identified as behaviors involving “study environment management” (McKeachie et al., 1986).

#### Surveyed beliefs and attitudes

As with students’ beliefs and attitudes toward learning, the second survey was organized in six scales meant to reveal some student beliefs associated with self-efficacy (Pajares, 1996; Schunk & DiBenedetto, 2014), growth mindset (Dweck, 2016), performance goals and learning goals (Kaplan & Maehr, 2007), control beliefs (Zimmerman & Schunk, 2006) and examination anxiety (Chapell et al., 2005). Since research has repeatedly reported associations between these constructs and learning techniques, as well as achievement (Dahl et al., 2005; Dupeyrat & Marine, 2001; Komarraju & Nadler, 2013; Lackey, 2014; Pintrich, 2000; Wolters et al., 1996), we expected to replicate these associations and aimed to analyze the existing relationships between beliefs and study techniques that are supported by cognitive research specifically.

For example, previous research has shown that students who adopt a learning goal orientation tend to use adaptive learning strategies more often, according to self-reports (e.g., Pintrich & DeGroot, 1990). However, research has also revealed that performance goals predict achievement more robustly than learning goals (Hulleman et al., 2010), and many studies have failed to find an association between grades and a learning goal orientation in school students, while the correlation with performance goals has usually emerged (Pintrich, 2000; Wolters, 2004). As Senko (2019) put it, “The most surprising and controversial finding in achievement goal research is that performance goals predict academic achievement more reliably than mastery goals.” This apparent contradiction highlights the possibility that supported learning strategies could not have a high influence on achievement as measured by grades.

Regarding self-efficacy beliefs, the association between self-efficacy and achievement is well documented (Pajares, 1996). While high self-efficacy may help students engage and persevere in doing what is needed to achieve, perhaps the role of achievement in modulating self-efficacy to confront future challenges provides a better explanation for their close association (Bandura, 1997; Schöber et al., 2018; Usher & Pajares, 2008). If self-efficacy is more a product of achievement than a cause, then helping students achieve would be the most effective method to enhance their self-efficacy and involve them in school activities. Therefore, if using appropriate studying techniques contributes to their achievement, teaching them how to learn and encouraging them to apply this knowledge should help enhance their self-efficacy and, consequently, their motivation. Some experimental studies have already provided evidence of the positive effect of teaching learning strategies on students’ self-efficacy (Lavasani et al., 2011). If this is the case, a correlation between supported learning strategies and self-efficacy should be expected. Given that self-efficacy is typically linked with control beliefs (Zimmerman & Schunk, 2006) and growth mindset (Dweck, 2000), these constructs should also show an association with supported learning techniques.

## Research hypotheses

Although we built the surveys according to the relative effectiveness that the study techniques have shown in experimental research, our hypothesis regarding the relationship between using effective techniques and school achievement (as measured by grades) expected associations to be weak in general, albeit stronger than for non-supported techniques. The rationale for this prediction is that we suspected that school assessments from which grades derive are often likely to be successfully completed by students even if they use ineffective techniques. It is important to highlight that techniques like rote learning, rereading, or massing are considered ineffective because they hardly contribute to transferable and durable learning, but in fact they can be quite effective in the short term and when examinations do not require students to demonstrate a high level of understanding. Likewise, it may be the case that study techniques which do not significantly relate to grades do foster more durable and transferable learning, but this learning is not revealed by school examinations. In other words, school grades may not equate to learning in the long term.

For example, one of the most supported learning strategies after more than a century of research, spaced practice, has yielded no significant correlation with achievement in previous studies with college students (e.g., Hartwig & Dunlosky, 2012). As mentioned earlier, this could be explained by the fact that students may be successful even if they mass their study sessions. In other words, examinations may not measure long-term, transferable learning—properties of learning usually enhanced by spaced practice (Carpenter et al., 2022)—but rather reflect the ability to remember information for a short period of time, something that cramming contributes to achieving quite well (Seabrook et al., 2005). In fact, back in 1988, Frank N. Dempster already wondered why such an important research outcome as the spacing effect had not become standard practice in education, so he suggested nine potential explanations. One such explanation referenced a number of studies which concluded that massed practice is equally as effective as spaced practice, and in some cases even more effective. These scenarios were referred to as "boundary conditions" (i.e., conditions in which spaced practice may no longer be effective). Specifically, Dempster concluded that massed practice works better than spaced practice on immediate examinations. In sum, we should expect that the associations between supported study techniques and achievement (as measured by examination grades) could be small or even nonexistent due to this disconnection between examination performance and long-lasting, transferable learning.

Our hypothesis involving students’ beliefs predicted higher positive correlations between study techniques supported by cognitive research and beliefs related to control, self-efficacy, learning goals, and growth mindset, compared to unsupported techniques. On the one hand, as mentioned earlier, previous research has suggested that students with higher levels of engagement in learning are more likely to use techniques that require higher levels of cognitive effort (Liem et al., 2008; Pintrich, 2000; Wolters et al., 1996), such as the techniques supported by research on cognitive psychology. On the other hand, effective techniques may help students succeed in school, which could increase their self-efficacy and control beliefs (Pekrun & Perry, 2014; Schöber et al., 2018; Usher & Pajares, 2008).

Finally, since previous research has reported that retrieval practice can reduce students’ test anxiety (Agarwal et al., 2014), we also expected to find a negative correlation between using techniques involving retrieval and reported anxiety levels.

## Method

### Procedure

Following approval from the Institutional Review Board (IRB), participant schools were recruited using personal communication. The researchers provided all students and families with informed consent documents to confirm their will to participate in the study. In particular, they were informed that participation in the research was optional and that the information collected by the researchers would not include personal data since a non-identifying, individually created alphanumeric code would be used to connect survey responses with school grades.

Participant students completed each online survey on two separate days (to avoid non-reflexive answers due to tiredness), in the same order—firstly, the survey on strategies, and secondly, the survey on attitudes and beliefs—, and during the class time usually devoted to discussing extracurricular topics with their school tutors. Non-participating students read or did homework during that period of time. At the end of the school year, schools provided the final grades of participating students properly coded for all the subjects they attended.

### Participants

The study sample consisted of 5063 secondary school students (grades 7th to 12th) from 27 schools widely distributed across the geography of Catalonia (the north-eastern region of Spain). Participant schools included urban schools from different districts of the city of Barcelona, as well as schools in towns and villages across Catalonia, which collectively provided a highly diverse sample in socioeconomic terms. Since only 4373 students completed the two surveys and identified their answers with a correct code, the rest of the answers were discarded. In addition, of all the grade sheets provided by the schools, a total of 921 did not include all the required information, so we discarded the answers of those students as well. In the end, the final sample totaled 3414 students.

### Instruments

As in previous studies, data collection was based on self-reports (except for grades, which were provided by schools). We created two surveys specifically for this study (Appendixs 1 and 2).

The learning techniques survey assessed how often students engaged in many of the study behaviors that have been previously described in literature and, in fact, its design was guided by instruments applied in previous research (e.g., Bartoszewski & Gurung, 2015; Blasiman et al., 2016; Gurung et al., 2010; Hartwig & Dunlosky, 2012; Karpicke et al., 2009; Kornell & Bjork, 2007; Taraban, 1999) and widely used tests such as LASSI (Weinstein et al., 1987). Each item assessed one of the many study behaviors that students usually report and was then classified into four types of techniques according to the underlying cognitive principles implied by each one: retrieval practice (eight items), distributed practice (ten items), focus (seven items), and elaboration (fifteen items). Since elaboration is a broad term, this category was also divided into three subcategories, grouping items related to processing information to organize it or paraphrase it (five items); items related to explicitly looking for relationships between new information and prior knowledge (four items); and, finally, items expressing the explicit intention to understand what is learned (six items). The latter subcategory included metacognitive behaviors related to seeking and monitoring understanding.

A fifth category grouped behaviors frequently reported by college students in previous studies irrespective of their degree of effectiveness according to research (Bartoszewski & Gurung, 2015; Blasiman et al., 2016; Hartwig & Dunlosky, 2012; Karpicke et al., 2009; Kornell & Bjork, 2007). All these behaviors imply techniques which share a low level of elaboration. These behaviors were organized in three groups: rote learning (two items), highlighting & copying (three items), and rereading (three items).

It is worth highlighting that the described categories were not defined to group behaviors that students usually combine and which should therefore correlate—somehow responding to a latent factor. Instead, they were arranged to consider whether those behaviors share the underlying principles that cognitive research suggests are effective. Indeed, the different study behaviors that students report need not be coherent with regard to these principles. For example, students may report to frequently engage in a behavior related to retrieval practice, such as testing themselves, but not engage in some other behavior that also implies retrieval, such as explaining the lesson to other people. Therefore, these categories follow a functional approach that could not be validated by factor analysis. Actually, LASSI, one of the most widely used instruments to assess student learning strategies (Cano, 2006; Weinstein et al., 1987), was also designed using a functional approach (Cano, 2006). As other researchers have pointed out, “reliance on factor analysis alone to validate scales of this type is not justifiable; it is equally important to maintain the conceptual clarity of the group of items” (Entwistle et al., 1991). Other researchers such as Bartoszewski and Gurung (2015) have used the same approach to group their survey items according to the study techniques that they involve (e.g., practice testing, self-explanation, rereading, etc.) and have analyzed their correlation with achievement. Ultimately, the aim is to analyze how the cognitive principles that underlie different behaviors are associated with achievement, even if students are not aware of them. That said, the items were grouped based on expert knowledge and following the same criteria used in previous studies. Additionally, we conducted a robustness analysis to corroborate that our results did not depend on an arbitrary grouping strategy of the items. For that purpose, we recomputed the analyses after randomly leaving one item out of each group and checked that this did not substantially alter the results.

The responses to the items were collected using a five-point Likert-type scale corresponding to how often students displayed the described study behavior (1- never; 2-hardly ever; 3-sometimes; 4-often; 5-always). Some items were reverse-scored since they described the opposite behavior that was intended to be measured (e.g., “I listen to music while I study,” as opposed to avoiding extraneous cognitive load). At the end of the survey, we further included a question asking students how helpful they thought self-testing was as a learning technique. This question offered three possible explanations as to why self-testing can be helpful, and students had to report their level of agreement or disagreement using the Likert-type scale described above. A final question asked students whether they had been taught how to learn or whether they had figured out how to do it by themselves. Appendix 1 includes a translated version of the survey from Catalan into English.

The second survey assessed several students’ beliefs and attitudes toward learning. The survey was initially designed based on expert knowledge, and most items in the survey were adapted from various existing instruments, such as the amply used MSLQ (Pintrich et al., 1993). The items were organized in six scales: the “self-efficacy” scale included six items assessing students’ confidence in their ability to successfully complete school tasks and assignments; the “learning goals” construct contained six items dealing with students’ interest in wanting to learn what they are taught; the “performance goals” scale consisted of four items that explored students’ emphasis on getting good grades; the “control beliefs” construct comprised four items that assessed the extent to which students believed that their success in school depends on causes they can control, such as effort or study techniques; the “growth mindset” scale included two items asking students whether they thought that the ability to achieve in school is malleable; and the “examination anxiety” scale included of five items that asked about their perceived level of stress when facing examinations.

In this case, we grouped the items assuming that they were measuring the same constructs, respectively, and therefore we tested the validity of the instrument by conducting a confirmatory factor analysis. To do that, we used a sample of 1 000 participants randomly selected from the total sample and conducted a confirmatory factor analysis for a model including the six latent factors. After some revisions during which we excluded certain items that were not contributing to the robustness of the model, the best model yielded a GFI of 0.980, a SRMR of 0.069, a RMSEA of 0.071, a CFI of 0.856, a TLI of 0.836 and a RNI of 0.856. Although these results are not optimal to conclude that the model represents the latent factor structure underlying the data, they are good enough for this kind of tests (Matsunaga, 2010). In fact, they are much better than the fit indices obtained by the authors of one of the most widely used instruments, the MSLQ (Pintrich et al., 1993). Besides, the scales showed an acceptable level of reliability using Cronbach’s alpha: α = 0.802 for “self-efficacy”; α = 0.789 for “learning goals”; α = 0.870 for “performance goals”; α = 0.756 for “control beliefs”; α = 0.637 for “growth mindset”; and α = 0.819 for “examination anxiety.”

The responses to the items were provided on a Likert-type scale with five levels of agreement or disagreement (1 strongly disagree to 5 strongly agree).

### Data analysis

Since we used the data from 1 000 participants to adjust and validate the instrument measuring students’ beliefs and attitudes toward learning, the following analyses were performed with the remaining data (n = 2 414).

For each survey, we conducted correlation analyses of each scale and subscale with (a) the students’ average grade, and (b) the scales and subscales of the other survey. We also conducted all pairwise contrasts between the eleven study techniques.

Some specific correlation coefficients were compared using the *cocor* package for R (Diedenhofen, 2015).

Finally, we used between-subjects t-tests to compare participants who answered they had received instructions on how to study to participants who answered they had not.

Since the sample was very large, to avoid Type-I errors we decided to: (a) correct all *P*-values using the Bonferroni method, and (b) interpret as significant only those values below a highly restrictive threshold (i.e., *P* < 0.001 after the correction).

## Results

### Prevalence of study techniques and beliefs

Table 1 summarizes the descriptive statistics (mean and standard deviation) for each scale and subscale from both surveys (see also the distribution of scores in Supplementary Information, Additional file 1). Data show that students reported using high elaboration techniques as much as rote learning. However, students placed highlighting-copying and rereading as their least preferred techniques.

**Table 1:** Mean and standard deviation for each scale and subscale in both surveys (n = 2414). Note: scores range from 1 to 5 points

	Mean	SD
*Study techniques*		
Focusing	3.790	0.640
Spacing	3.130	0.450
Low elaboration	3.235	0.621
· Rereading	3.088	0.685
· Highlighting-copying	2.973	0.957
· Rote learning	3.593	0.955
High elaboration	3.523	0.626
· Monitoring comprehension	3.820	0.703
· Associating	3.343	0.890
· Processing	3.310	0.777
Retrieval practice	3.213	0.738
*Beliefs & attitudes*		
Control beliefs	3.981	0.713
Growth mindset	3.863	0.785
Learning goals	3.295	0.793
Performance goals	4.246	0.773
Examination anxiety	3.146	0.918
Self-efficacy	3.658	0.692

Spaced practice and retrieval practice were among the least used techniques, which means that students often mass their study time and do not usually engage in testing themselves. Actually, the question regarding the technique that involves preparing questions for self-assessment (e.g., flashcards) had a very low mean score (2.512), only offset by other low elaboration retrieval techniques with higher mean scores such as rehearsing (3.694).

Nearly all the paired-sample t-tests for the differences between study techniques (Appendix 3) were statistically significant (*P* < 0.001), as was to be expected given the large sample, except for the following pairs: “rote learning” and “high elaboration,” “retrieval practice” and “spacing,” “rereading” and “spacing,” “retrieval practice” and “low elaboration,” “associating” and “processing,” and “focusing” and “monitoring comprehension.”

Regarding the management of the study setting, it is worth noting that 25.31% of the students admitted to always or almost always studying while listening to music, and 24.25% reported similar behavior regarding the habit of checking their cell phones during the study session (for reasons unrelated to the learning goals). The correlation between these two types of behavior that introduce extraneous cognitive load in the study task was weak (r = 0.205). Besides, only 3.84% of students reported having the TV on in the background while studying.

According to the students’ reports in the second survey, a performance goal orientation was more common than a learning goal orientation.

### Associations with academic achievement

**Fig. 1:**
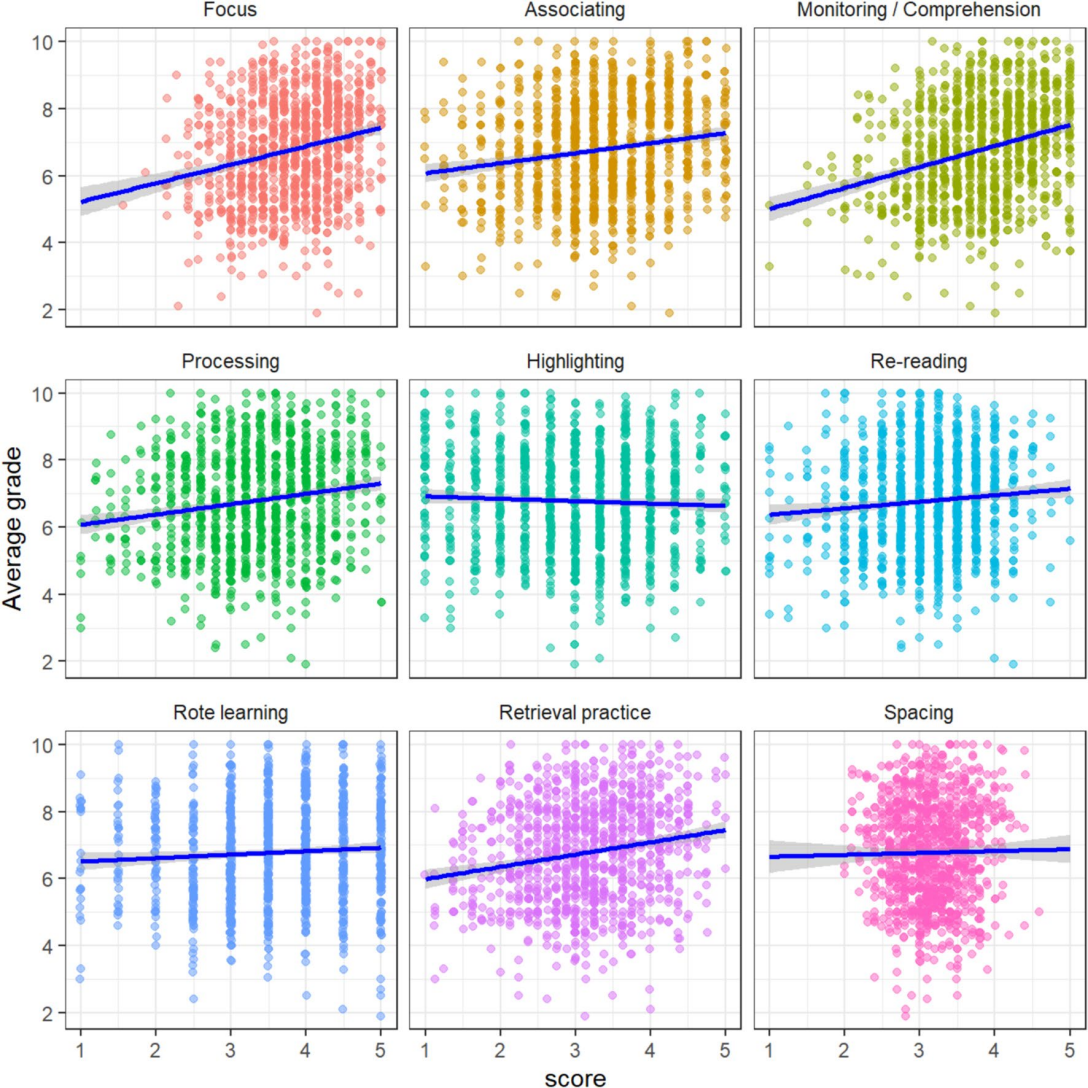
Scatterplots with the correlations between the study techniques and academic achievement

Table 2 shows Pearson’s correlations between scales and subscales in each survey and achievement (as measured by average grade). Regarding the data from the study techniques survey (Fig. 1), although all correlations were low (r < 0.3) and positive (except for the subscale “highlighting-copying”), there was a remarkable difference between techniques such as “spacing” or “low elaboration,” which showed correlation scores below 0.1, and “high elaboration,” “retrieval practice,” or “focusing,” which all yielded Pearson’s r coefficients above 0.2. Actually, the associations between grades and “spacing” or “low elaboration” techniques were not statistically significant, even in spite of the large sample. All other associations were significant for *P* < 0.001 (after the Bonferroni correction). It is important to note that the specific items asking about the habit of listening to music or checking the Internet during study time yielded significant, small, negative correlations (r = − 0.118 and r = − 0.113, respectively). The highest correlation with grades (r = 0.300) was achieved when combining all research-supported techniques except for distributed practice.

**Table 2:** Pearson’s correlations with average grades (n = 2414; k = 17). k is the number of family-wise contrasts used for the Bonferroni correction. Corrected *P*-values above 1 are shown as 1. Asterisks indicate statistically significant associations (*P*-values corrected < 0.001)

	Pearson's r	*P*-value (corrected)
*Study techniques*		
Focusing	0.213*	< 0.001
Spacing	0.043	0.595
Low elaboration	0.054	0.136
· Rereading	0.029	1
· Highlighting-copying	− 0.011	1
· Rote learning	0.048	0.323
High elaboration	0.264*	< 0.001
· Monitoring comprehension	0.270*	< 0.001
· Associating	0.164*	< 0.001
· Processing	0.195*	< 0.001
Retrieval practice	0.224*	< 0.001
*Beliefs & attitudes*		
Control beliefs	0.221*	< 0.001
Growth mindset	0.200*	< 0.001
Learning goals	0.125*	< 0.001
Performance goals	0.288*	< 0.001
Examination anxiety	− 0.192*	< 0.001
Self-efficacy	0.555*	< 0.001

Analyses of the associations between beliefs and grades (Table 2) indicate that the highest correlation (r > 0.5) belonged to “self-efficacy,” followed by a much lower, positive correlation for “performance goals” (r = 0.288), “control beliefs” (r = 0.221), “growth mindset” (r = 0.200), and “learning goals” (r = 0.125). Additionally, “examination anxiety” showed a weak and negative correlation with achievement (r = − 0.192). All associations were statistically significant (with corrected *P* < 0.001).

### Associations between study techniques

We looked for correlations between high elaboration and low elaboration techniques (Table 3). Remarkably, the association between elaborative and non-elaborative techniques was quite relevant (r = 0.495, *P*-value < 0.001). When the subscales were compared, they all yielded significant correlations (*P*-value < 0.001) with Pearson’s coefficients between 0.137 and 0.503. The higher correlation was between highlighting-copying and information-processing techniques, such as summarization or conceptual mapping (r = 0.503).

**Table 3:** Pearson’s correlations between study techniques (*P*-values corrected: *P < 0.001; n = 2414; k = 16)

	High Elaboration	Monitoring Comprehension	Associating	Processing
Low Elaboration	0.495*	0.394*	0.285*	0.508*
Rereading	0.354*	0.313*	0.227*	0.309*
Highlighting-copying	0.393*	0.218*	0.229*	0.503*
Rote learning	0.263*	0.254*	0.137*	0.234*

### Associations between beliefs

Table 4 contains Pearson’s correlations between beliefs and attitudes toward learning obtained from the second survey. It can be observed that the association between the two types of goals was small but positive (r = 0.289).

**Table 4:** Pearson’s correlations between beliefs and attitudes toward learning (*P*-values corrected: *P < 0.001; n = 2414; k = 15)

Beliefs & Attitudes	Growth mindset	Learning goals	Performance goals	Examination anxiety	Self-efficacy
Control beliefs	0.367*	0.341*	0.327*	0.038	0.323*
Growth mindset	–	0.356*	0.314*	− 0.026	0.355*
Learning goals	–	–	0.289*	0.022	0.363*
Performance goals	–	–	–	0.143*	0.408*
Examination anxiety	–	–	–	–	− 0.294*

“Self-efficacy” had the highest correlation with “performance goals” (r = 0.408), followed by “learning goals” (r = 0.363), “growth mindset” (r = 0.355), and “control beliefs” (r = 0.323). Its association with “examination anxiety” was also moderate, but negative (r = − 0.294).

The construct “learning goals” positively correlated with “growth mindset” (r = 0.356). The correlation between “performance goals” and “growth mindset” was also positive and moderate (r = 0.314).

The “control beliefs” scale yielded positive, moderate correlations with all other scales (r > 0.3), except for “examination anxiety,” which was close to zero and nonsignificant (r = 0.038).

Finally, “examination anxiety” yielded a weak, positive correlation with “performance goals” (r = 0.143), but no significant correlation with “learning goals.” It did not show a significant correlation with “growth mindset” either.

### Associations between study techniques and beliefs

Table 5 shows the results after cross-analyzing the associations between the scales from both surveys.

**Table 5:** Pearson’s correlations between study techniques and beliefs or attitudes toward learning (*P < 0.001; n = 2414; k = 30)

	Control beliefs	Growth mindset	Learning goals	Performance goals	Examination anxiety	Self-efficacy
Focusing	0.224*	0.206*	0.173*	0.216*	− 0.109*	0.235*
Spacing	0.125*	0.078	0.138*	0.062	− 0.115*	0.050
Low elaboration	0.196*	0.143*	0.162*	0.211*	0.252*	0.059
High elaboration	0.321*	0.310*	0.378*	0.340*	0.104	0.322*
Retrieval practice	0.294*	0.256*	0.359*	0.320*	0.149*	0.274*

In general, “high elaboration” techniques and “retrieval practice” clearly yielded higher associations with all constructs than “low elaboration” techniques, except for “examination anxiety,” which had no significant correlation with “high elaboration” and a weaker association with “retrieval practice” than “low elaboration” techniques. Remarkably, “low elaboration” techniques did not show a significant association with “self-efficacy,” while “high elaboration,” “retrieval practice” and “focusing” yielded Pearson’s coefficients of correlation higher than 0.2.

As with the two types of goals, associations with “high elaboration” techniques and “retrieval practice” were quite similar (no significant differences were found between their correlation coefficients), but there were clear differences when compared to the other techniques. Indeed, while “performance goals” yielded higher correlations with “low elaboration” techniques and “focusing” than “learning goals” did, the latter were more associated with “spaced practice.”

“Spacing” only yielded significant, positive associations with “control beliefs” and “learning goals,” although they were weak (r < 0.2). Interestingly, it also yielded a significant, negative correlation with “examination anxiety” (r = − 0.115). All associations with “examination anxiety” were positive, except for “spacing” and “focusing.” The higher correlation with “examination anxiety” (r > 0.2) was with “low elaboration” techniques (r = 0.252).

### Other results

As with the items that intended to uncover students’ beliefs about self-testing, the results showed that students using this technique thought it was useful to check whether they had learned what they had studied (mean = 4.418) and to find out what needed to be reviewed (mean = 4.314). Respondents showed less support for the idea that self-testing strengthens learning implicitly (mean = 4.088).

Finally, 77.96% of students reported not having received any guidelines about how to study. Analysis of the possible differences between students based on their responses to this question yielded only one significant result regarding learning techniques: students who reported having been taught how to study were more likely to apply spaced practice than those who did not (Cohen’s d = 0.258; corrected *P*-value < 0.001). However, their average grades were also lower (d = − 0.345; corrected *P-value* < 0.001).

## Discussion

### Prevalence and associations between study techniques

This study simultaneously assessed the extent to which secondary school students use techniques that are supported by cognitive research as well as other frequently used techniques previously reported in other studies.

Through anecdotal observation, one may be under the impression that school students mainly rely on rote learning, but our data show that they are aware of the importance of understanding what is being learned. Paradoxically as it may sound, students admit using rote learning as much as they report their willingness to comprehend the study material. In fact, elaborative techniques such as summarization or self-explanation were more frequently reported than “low elaboration” techniques, such as highlighting or rereading, which showed the lowest prevalence scores. This could mean that although students realize the convenience of learning with understanding, they heavily rely on rote learning because they do not usually have the time and opportunities to understand what they learn, and so they choose to memorize it. In addition, students may possibly rely mainly on rote memorization because they are aware that, at the end of the day, this technique is effective for passing school examinations. In any case, the moderate correlations between “low elaboration” and “high elaboration” techniques reveal that even students who rely on effective techniques usually apply less effective techniques, too. Nevertheless, the remarkable correlation (r = 0.503) between “highlighting-copying” and “processing” (i.e., summarization, conceptual mapping, etc.) could be explained by the fact that students might create summaries by first highlighting texts and then copying the sentences, instead of paraphrasing. If this is the case, summarization could lose efficacy as an information-processing technique (Glover et al., 1981; Thai, 2021), something that could explain why Dunlosky et al. (2013) rated summarization as a low utility technique despite the degree of elaboration that it should ideally involve.

Counter to the results from recent studies with university students (e.g., Bartoszewski & Gurung, 2015), retrieval practice was among the least used strategies, especially elaborative retrieval techniques such as preparing questions for self-testing. Rehearsing after reading, on the other hand, was the most frequent application of retrieval practice. This would be consistent with the fact that rote learning was among the most preferred approaches to studying, and so retrieval would be often used as a memorizing strategy. The fact that retrieval practice would be more common in college students than in school students could be explained by the selection process used to gain access to university, which benefits students with higher grades. As mentioned later in Discussion, the data we collected indicates that retrieval practice is more frequent among students with higher grades. Hence, this technique seems to be more frequent among the college student population simply because students who continue their higher education at university are more likely to use it than those who follow other paths. Interestingly, a significant number of students using self-testing thought that it contributed to memory retention, although the main reason for using it was to make sure that they remembered the study material, which is usually the main role that college students (and teachers) believe testing has (Hartwig & Dunslosky, 2012; Roediger & Karpicke, 2006).

Another highly recommended strategy that stayed on the lower end of our list as one of the less prevalent studying habits is spaced practice. But this comes as no surprise; previous data have shown that, even in higher education settings, students are prone to mass their study sessions, especially on the days right before examinations (e.g., Blasiman et al., 2016).

Regarding the management of the study setting to keep distractions away, the fact that our student sample reported a preference to study in a quiet environment aligns well with the recommendations put forth by cognitive load theory, although 1 out of 4 students admitted to always or almost always studying while listening to music. Of course, when interpreting data regarding the study environment, it is necessary to consider that oftentimes students do not have the opportunity to choose a quieter environment because of their home conditions. Then, students may prefer to listen to music while studying to mask a noisy environment. While background music could be deleterious for study performance (Anderson & Fuller, 2010; Perham & Currie, 2014), it might be better than noise, especially if the latter includes intelligible speaking (Keus van de Poll et al., 2014).

Finally, it is worth mentioning that 77.96% of the students in our sample reported to not have been taught how to study—a result very close to that obtained by Kornell and Bjork (2007) with college students—which brings to mind the paradox that Don Norman already lamented in 1980: “It is strange that we expect students to learn yet seldom teach them about learning.”

### Prevalence and associations between beliefs and attitudes toward learning

As far as beliefs and attitudes toward learning are concerned, our data show that the secondary school students in our sample clearly pursue performance goals over learning goals. This suggests that grades rather than learning are their first priority. According to research on achievement goal theory, there is a positive association between the goal structure that students perceive as being emphasized in their environment and the personal goal orientation that they adopt (Wolters, 2004). In light of this, it could be suggested that our student sample is influenced by a learning culture that prioritizes performance over competence. This influence could be implicit, meaning that students may perceive that school achievement is possible without gaining deep, long-lasting learning, and hence assume that that is what is expected from them. Since we found a weak positive correlation between the two types of goals (r = 0.289), this means that they are not mutually exclusive, but that students who strive for learning goals are more likely to pursue performance goals, too. This is consistent with the literature on students’ goals, which suggests that students adopt or pursue multiple types of goals within any academic setting (Niemivirta, 2019; Valle et al., 2003; Wolters, 2004).

That said, it may be appropriate to reflect here on the concerning fact that students may see performance goals (such as achieving high grades) and learning goals (such as gaining knowledge and understanding) as independent of each other. In other words, although students' performance in school assessments should act as evidence to infer their learning, the way learning is assessed in school relies on a type of performance that is not appropriate to provide evidence of long-lasting, transferable learning (Soderstrom & Bjork, 2015). Furthermore, the fact that the correlation between students’ learning goals and performance goals is not high indicates that students are probably aware of this. Therefore, one could expect the studying behaviors associated with each type of goal to be different. This is discussed further later in the article, where we address the correlations between goals and study techniques found in our data.

Our results also reveal that the students in our sample hold ideas that are more aligned with a growth mindset than a fixed mindset. Proponents of mindset theory claim that holding a growth mindset is related to a learning goals orientation, that is, students with growth mindsets focus on learning and embrace challenges (Braten & Stromso, 2004). On the contrary, students who hold a fixed mindset are more centered in performance goals since they believe that academic outcomes reflect abilities they cannot change, and so they focus on appearing talented (Dweck, 2016). According to our data, correlations between the two types of goal orientation and “growth mindset” were quite similar (r > 0.3). Although “learning goals” yielded a slightly higher correlation coefficient, when both correlations were compared using the *cocor* package for R the difference turned out to be nonsignificant. Therefore, our results were not consistent with the theory. As other researchers have pointed out, the distinctive relationship between beliefs about ability and goal orientations is not clear, and in any case, it is usually too weak to consider them part of the same constructs (Burnette et al., 2013).

It is worth noting that “growth mindset” did not yield a significant association with “examination anxiety,” which is not consistent with the idea that a “growth mindset” (i.e., believing that the ability to do well in school is malleable and can be changed) helps students face school challenges because they do not see them as situations that could reveal defects that define them as learners, rather than mere evidence of their progress (Dweck, 2016). Actually, “beliefs of control” did not significantly correlate with “examination anxiety” either, although according to the control-value theory of achievement emotions (Pekrun & Perry, 2014) a negative correlation between them should have been expected. Although “growth mindset” and “beliefs of control” showed similar correlations with the rest, which is coherent given their relatedness, the correlation between them was only moderate (r = 0.367).

Self-efficacy beliefs in our sample tended to be positive. Interestingly, “growth mindset” was positively associated with “self-efficacy” (r = 0.355). This result is consistent with claims by mindset theorists (Dweck, 2000). It may well be that the idea that achievement is not heavily determined by one’s innate ability, but that it greatly depends on work, contributes to enhancing self-efficacy. Alternatively, the accomplishments that contribute to self-efficacy may also contribute to developing beliefs closer to a growth mindset, while failure leads to ideas typical of a fixed mindset, like underestimating effort as a means to achieve (Tek et al., 2018). Whatever the reason for this association is, our data are consistent with data from other studies (e.g., Komarraju & Nadler, 2013; McWilliams, 2015).

The associations between self-efficacy and the two types of goals are probably more interesting. Indeed, both “learning goals” and “performance goals” showed a relevant association with “self-efficacy” (r = 0.363 and r = 0.408, respectively), and this difference was not statistically significant (z = 2.07, *P* = 0.0380). This leads us to speculate that students with higher self-efficacy are more prone to pursuing learning goals because they are more confident about achieving performance goals. In this regard, previous studies have found that high self-efficacy students usually place greater emphasis on both performance and learning goals (e.g., Komarraju & Nadler, 2013). What comes as no surprise is the negative association between “self-efficacy” and “examination anxiety” since it makes sense to expect that the more confident students are to achieve, the lower their level of examination anxiety, which is usually related to the fear of not achieving (Zeidner, 1998).

### Associations between study techniques and achievement

The first result that stands out in our data is the fact that spaced practice yielded no significant correlation with school achievement. However, as we had already hypothesized, this could be explained by the fact that students may be successful at school even if they mass their study sessions. This is related to the concept of “boundary conditions” suggested by Dempster (1988): scenarios in which spaced practice is not more effective than massing, such as dealing with immediate examinations, since spacing contributes to learning qualities (i.e., durability and transfer) typically not evaluated in school examinations.

The same notion of “boundary conditions” may in part explain why other well-supported study strategies, such as retrieval practice, yielded only weak correlations (r ≈ 0.2) with school achievement. Indeed, many experiments conducted both in the laboratory and in the classroom using real school material have shown that the effect of retrieval practice is usually salient when performance is measured in the long term, rather than right after the study session. In the short term, retrieval may be as effective as other strategies such as rereading (Roediger & Karpicke, 2006). Therefore, as was the case with massing versus spacing study, if students can obtain successful school grades by using strategies that are effective in the short term but do not especially contribute to long-term retention, it is plausible to assume that well-supported strategies will not contribute to school success as much as they theoretically could. In any case, since transient learning is not what we seek in education, an urgent reflection is needed regarding the way in which learning is being assessed in schools and how it is actually related to achievement.

Despite the fact that school examinations may not be assessing the qualities of learning that research-supported study strategies usually enhance (which should be the goals of education), our data reflects that techniques such as retrieval practice and elaboration are more clearly associated with school achievement than non-supported techniques. Actually, it is worth stressing that techniques such as highlighting or copying notes or the textbook yielded no significant correlation with achievement in spite of the large sample size. Regarding this, it is always important to keep in mind that correlation does not imply causation, but also that, in this scenario, absence of correlation may imply lack of causation. In other words, while the results of this study do not allow us to conclude that correlation between retrieval or elaboration and achievement is due to the contribution of these techniques to examination performance, the lack of correlation found in highlighting and copying suggests that such techniques are not contributing to it. Therefore, students would be better off replacing these behaviors with others more likely to help them.

Ultimately, the fact that positive correlations between research-supported techniques and achievement are clearly higher (and significant) than “low elaboration” techniques is consistent with the assumption that supported techniques may be contributing to school success. If we combine the three scales comprising “high elaboration” techniques, “retrieval practice” and “focus,” the correlation adds up to a Pearson’s coefficient of r = 0.300, while non-supported techniques only reach a nonsignificant correlation of r = 0.054. This means that according to our data, study behaviors supported by cognitive science could explain 9% of school achievement variance. However, it is possible that students with higher cognitive ability tend to use more cognitively demanding strategies, which may explain the correlation with achievement, at least partially. Further research controlling for cognitive ability would be helpful to better uncover the contribution of study techniques to achievement in secondary school.

Finally, the correlations between grades and techniques involving the management of extraneous cognitive load during the study task are consistent with the hypothesis that a quiet study environment would benefit learning. Of special interest is the significant, negative correlation between the item asking specifically about listening to music while studying and students’ grades, given its consistency with the literature on the deleterious effects of music on students’ performance (Anderson & Fuller, 2010; Perham & Currie, 2014), something which is often ignored or discredited by students who share that habit.

### Associations between beliefs or attitudes and achievement

As to the associations between students’ beliefs or attitudes and achievement, our hypothesis regarding a relevant association between self-efficacy and grades was confirmed (r = 0.555).

Likewise, the scales for “growth mindset” and “control beliefs” yielded positive correlations with achievement, albeit small. Just as self-efficacy beliefs may be a consequence of achievement more than a cause (Schöber et al., 2018; Usher & Pajares, 2008), it is also reasonable here to assume that achievement has an influence on students’ control beliefs and mindset. If students achieve their goals after trying, then it is likely that they attribute their success to that effort (at least in part), which is typical of a growth mindset. However, if they fail despite having tried, it is logical that students’ control beliefs suffer and for them to conclude that school is just not for them, a distinctive position of those who adopt a fixed mindset (Tek et al., 2018). Additionally, it may well be that believing in the need for working hard to achieve goals prevents students from falling into self-fulfilling prophecies, which occur when they believe that effort is not decisive for achievement (but that ability is) and hence they choose not to make it, leading them to fail just because of that. In any case, research on mindset interventions that aim to impact school achievement suggests that achievement is unlikely to be the consequence of mindset beliefs (Macnamara & Burgoyne, 2022).

As with students’ goals, “performance goals” yielded a much stronger correlation (r = 0.288) with achievement than “learning goals” (r = 0.125), and this difference was statistically significant (z = 6.93, *P* < 0.001). This does in fact not come as a surprise since research has shown that performance goals predict achievement more robustly than learning goals (Hulleman et al., 2010; Pintrich, 2000). Although different hypotheses have been put forward to explain this phenomenon (Senko, 2019), in our opinion school assessments do not often differentiate between deep, long-term learning and shallow, ephemeral learning, and therefore the distinctive behaviors that could be related to learning goals, which are aimed at achieving a better competence, would only have a negligible effect on school achievement (Wolters, 2004). For example, our data showed that learning goals were more strongly associated with distributed practice, but distributed practice did not correlate with achievement, although this learning strategy usually fosters more transferable, long-lasting learning. Additionally, some researchers have suggested that students who report a learning goal orientation may dedicate a disproportionate amount of their study time to content that they find personally intriguing, rather than to material that is less engaging, and that this behavior might compromise their academic achievement if it leads them to neglect any school content that they consider uninteresting (Senko & Miles, 2008; Senko et al., 2013).

### Associations between study techniques and beliefs or attitudes toward learning

Previous research has suggested that students with higher levels of engagement in learning are more likely to use techniques which require higher levels of cognitive effort (McWhaw & Abrami, 2001; Meece et al., 1988; Pintrich, 2000; Ross et al., 2022; Wolters et al., 1996). However, this study did not find a significant difference between the association of “high elaboration” or “retrieval” techniques with “learning goals” compared to “performance goals.” Interestingly, “performance goals” were more strongly associated with “low elaboration” techniques than were “learning goals” (although the difference was not significant: z = 2.07, *P* = 0.0386), which suggests that this type of goal orientation makes students more prone to using less demanding strategies that may lead to examination success but that do not typically foster long-term learning. Nevertheless, the association with “spaced practice” was higher for “learning goals,” although the size of the effect was very small.

It is interesting to note that “growth mindset” was positively associated with all study techniques (except spacing), but especially with “high elaboration” techniques (r = 0.310) and retrieval practice (r = 0.256). Control beliefs showed the same trend, except for spacing. These correlations may indicate that believing in the relevance of effort to achieve a goal is associated with making that effort. After all, high elaboration techniques and retrieval practice require a higher cognitive effort (Bjork & Bjork, 2011).

As with self-efficacy, it is worth noting its lack of association with study techniques not supported by research. This means that these types of techniques are distributed among students regardless of their level of self-efficacy, but also that these techniques may not influence self-efficacy at all. As has been mentioned before, self-efficacy is believed to be positively influenced by positive outcomes and it may then be more related to strategies that help students succeed. This is consistent with the fact that techniques supported by research (except distributed practice) showed positive although weak correlations with self-efficacy (above 0.2), while unsupported techniques did not correlate. Again, the fact that the correlations were small and that distributed practice was not associated with self-efficacy may be due to the “boundary conditions” referred to by Dempster (1988).

Since previous experimental research has reported that retrieval practice may help students regulate anxiety (Agarwal et al., 2014), we expected to find a negative correlation between using techniques involving retrieval and reported anxiety levels. However, that was not the case. Examination anxiety positively correlated with all the techniques except for “focusing” and “spaced practice.” Nevertheless, its association with retrieval practice was weaker than that with “low elaboration” techniques and nonsignificant with elaborative techniques, suggesting that students with higher reported levels of examination anxiety are more likely to be using unsupported techniques and study in environments more prone to distractions. This is consistent with research suggesting that adaptive study skills may protect against test anxiety, something that has been experimentally supported by interventions for teaching study skills to elementary school students (Beidel et al., 1999). Interestingly, “spaced practice” yielded a negative correlation with “examination anxiety,” which could be explained by the fact that spacing may help students feel they are better prepared to face examinations. Of course, further research would be needed to contrast this hypothesis.

### Conclusions and further research

This study has revealed that study techniques supported by cognitive research show a higher association with school achievement than other non-supported, frequently used study techniques. It has also shown positive correlations between these study techniques and important constructs such as achievement goals (both performance and learning goals), self-efficacy, control beliefs, and growth mindset. Nevertheless, our data has also reflected that the association between learning behaviors and school achievement is weak, meaning that if there is a contribution of learning techniques to school achievement, this must be modest. Additionally, our results have reiterated that one of the most robust learning strategies, spaced practice, shows no association with school achievement, which puts into question the way learning is assessed in schools.

Regarding the limitations of this study, it is important to point out that we did not obtain any measure of cognitive ability that we could use to control this variable. Had we done so, we could have improved our analysis of the extent to which the relationship between study strategies and achievement is independent of cognitive ability. As we already mentioned, it is possible that students in our sample with higher cognitive ability tend to use more elaborative strategies, and hence the association between these strategies and achievement may be in part explained by this ability. In addition, it would have been interesting to reveal whether the association between achievement and strategies depends on cognitive ability. For example, do students with higher cognitive ability exhibit a weaker association between avoiding extraneous cognitive load and achievement? This would make sense if we consider that the greater the working memory capacity, the smaller the impact of superfluous cognitive load (Christopher & Shelton, 2017).

An idea for further research is to compare the results of our study to a sample of higher education students responding to the same surveys. We hypothesize that the association between research-supported learning techniques and achievement might increase, especially in the case of distributed practice. The rationale for this is that in higher levels of education, given the amount and complexity of the materials assessed in examinations, students could benefit more from using effective techniques. However, previous correlational studies with college students have not found associations between the use of distributed practice and academic achievement (e.g., Hartwig & Dunlosky, 2012).

A strength of this study is the size and heterogeneity of the sample. Most studies collected data from relatively uniform samples (often psychology students from the same college) or students from the same school or district. In our case, we administered surveys to students from 27 schools with very diverse student populations in socioeconomic terms and from a large geographical region (Catalonia), increasing the representativeness of our sample. In any case, a relevant concern with these quantitative approaches to students’ use of learning strategies, such as that conducted in preceding studies (e.g., Bartoszewski & Gurung, 2015; Hartwig & Dunlosky, 2012), is that they are based on unguided self-reports, which could be influenced by student biases. For example, students who are concerned with appearing competent may be more likely to overstate their use of learning techniques. This is an inherent limitation of these type of studies that is difficult to avoid, especially if ecological validity is intended to be preserved. In any case, other methodological approaches should be explored in the future.

As a final note, we would like to express that although we have been referring to the magnitude of the associations (measured by Pearson’s r) using the commonly applied criteria suggested by Cohen (1988) (i.e., *small, low*, or *weak* for r < 0.3, *medium* or *modest* for 0.3 < r < 0.5, and *large* for r > 0.5), a correlation coefficient larger than 0.2 should not be underrated considering the consequences that this effect size would have in school achievement, especially taking into account its cumulative effect (Funder & Ozer, 2019).

### Supplementary Information

**Additional file 1. **Distribution of scores: prevalence of use of the study techniques and prevalence of beliefs towards learning.

## Data Availability

The datasets generated and analyzed during the current study are available in the Mendeley repository, https://data.mendeley.com/preview/ddhvjyhgkk?a=914c7b57-f70e-4d7b-934d-49269469bfff.

## Appendix 1: Studying techniques survey

**Table:**

ID	
	When I have an examination one week ahead…
T1	I only study the day before the examination
T2	I only study the two days before the examination
T3	I study every day I can before the examination and review each day
T4	I don't study
	When I have an examination the next day…
T5	I stay up studying late into the night
T6	I wake up very early in the morning to review
	When I study…
T7	I highlight what I find most important
T8	I try to learn the content verbatim
T9	I recite what I have read multiple times
T10	I try to understand what I learn
T11	I come up with examples related to what I'm learning
T12	I try to explain the concepts in my own words
T13	I try to relate what I'm learning to things I already know
T14	I associate what I study with mental images
T15	After reading, I explain to myself what I have read
T16	If I don't understand something, I reread it to try to understand it
T17	I seek help when I have doubts
T18	I prepare questions and I test myself
T19	I ask someone to ask me questions
T20	I copy from the book or rewrite my notes
T21	I write summaries
T22	I create concept maps or diagrams
T23	I explain the lesson to other classmates or people
T24	When I need to learn a list of words, I come up with ways to remember them
T25	I like studying with other classmates
	To review…
T26	I review the lesson right after finishing studying it
T27	I review the lesson one or several days after studying it
T28	I choose not to review
	When I review…
T29	I test myself by trying to remember what I learned before revising it
T30	I don't test myself, but I reread the lesson again (once or multiple times)
T31	I review the activities without redoing them
T32	I only reread what I highlighted
T33	I reread everything that I feel I don't know well
T34	I identify my weak points and reinforce them
T35	I ask for help if there's something I don't understand
	When I study a subject that requires learning procedures (problems, etc.)…
T36	I review the way I solved previous exercises or the solution provided by the teacher
T37	I redo exercises I've already done without looking at the solution until I finish them
T38	I attempt new exercises without looking at the solution until I finish them
	If I realize that I already know something…
T39	I continue practicing it in that moment to consolidate it
T40	I wait for some time before continuing to practice
	While I study…
T41	I place myself in a quiet place
T42	I place myself in a place where I am alone
T43	I listen to music
T44	I turn on the television
T45	I keep checking my phone for unrelated things to what I'm studying
T46	I keep checking the internet for unrelated things to what I'm studying
	Testing myself while studying serves to…
T47	Check if I learned what I studied
T48	Facilitate the consolidation of what I have learned in my memory
T49	Identify my weak points to review them
T50	Would you say that you study in a certain way because someone taught you to study that way?
	Yes, someone taught me how to study
	No, I study in the way that seems best to me

Scales and subscales	# items	Items ID
Focusing	7	T25 inv, T41, T42, T43 inv, T44 inv, T45 inv, T46 inv
Spacing	10	T1 inv, T2 inv,T3, T5 inv, T6 inv, T26 inv, T27, T28 inv, T39 inv, T40
Low elaboration	7	
· Rereading	3	T7, T20, T32
· Highlighting-copying	3	T30, T31, T32, T36
· Rote learning	2	T8, T9
High elaboration	15	
· Monitoring comprehension	6	T10, T16, T17, T33, T34, T35
· Associating	4	T11, T13, T14, T24
· Processing	5	T12, T15, T21, T22, T23
Retrieval practice	8	T9, T15, T18, T19, T23, T29, T37, T38

## Appendix 2: Beliefs and attitudes survey

**Table:**

	As a student, I generally believe that…
B01	I can learn and master the skills taught at school
B02	I will receive good grades this year
B03	I can overcome any academic challenge
	In general, if I study, it's because…
B04	I care about my grades
B05	I am interested in what I learn
B06	I believe what I learn is important for my future
B07	I enjoy learning
	Regarding examinations…
B08	I get very nervous when I have a scheduled examination
B09	While studying for an examination, I can't stop thinking about how I will do
B10	I get very nervous while taking an examination
B11	While taking an examination, my mind goes blank
B12	While taking an examination, I can't stop thinking about the grade
	Regarding the subject of Spanish Language…
B13	I believe I can get a good grade
B14	I study because I care about my grades
B15	I study because I am interested in what I learn
	Regarding the subject of Mathematics…
B16	I believe I can get a good grade
B17	I study because I care about my grades
B18	I study because I am interested in what I learn
	Regarding the subject of Social Sciences…
B19	I believe I can get a good grade
B20	I study because I care about my grades
B21	I study because I am interested in what I learn
	To achieve good results in school, it is very important…
B22	The effort we put into studying
B23	The time we dedicate to studying
B24	The way we study
B25	The way we plan our tasks
	In my opinion…
B26	Our academic ability is an innate skill that we cannot change
B27	With work, we can improve our academic ability

Scales	# items	Items IDs
Beliefs of control	4	B22, B23, B24, B25
Learning goals	6	B05, B06, B07, B14, B17, B20
Performance goals	4	B04, B13, B16, B19
Examination anxiety	5	B08, B09, B10, B11, B12
Self-efficacy	6	B01, B02, B03, B15, B18, B21
Growth mindset	2	B26_inv, B27

## Appendix 3: Post Hoc comparisons-study techniques

**Table:**

Measure 1	Measure 2	t	df	p (corr)	Mean Difference	SE Difference
Focus	Spacing	46.348	2413	< 0.001	0.660	0.014
Focus	Low Elaboration	31.630	2413	< 0.001	0.555	0.018
Focus	Low-Highlighting	34.963	2413	< 0.001	0.817	0.023
Focus	Low-Rereading	36.817	2413	< 0.001	0.702	0.019
Focus	Low-Rote learning	8.782	2413	< 0.001	0.196	0.022
Focus	High Elaboration	16.591	2413	< 0.001	0.267	0.016
Focus	High-M. comprehension	− 1.882	2413	0.060	− 0.031	0.016
Focus	High-Associating	20.734	2413	< 0.001	0.447	0.022
Focus	High-Processing	25.384	2413	< 0.001	0.480	0.019
Focus	Retrieval practice	32.015	2413	< 0.001	0.577	0.018
Spacing	Low Elaboration	− 6.371	2413	< 0.001	− 0.104	0.016
Spacing	Low-Highlighting	7.132	2413	< 0.001	0.158	0.022
Spacing	Low-Rereading	2.412	2413	0.88	0.042	0.018
Spacing	Low-Rote learning	− 20.963	2413	< 0.001	− 0.463	0.022
Spacing	High Elaboration	− 25.368	2413	< 0.001	− 0.393	0.015
Spacing	High-M. comprehension	− 42.812	2413	< 0.001	− 0.690	0.016
Spacing	High-Associating	− 10.295	2413	< 0.001	− 0.213	0.021
Spacing	High-Processing	− 9.809	2413	< 0.001	− 0.180	0.018
Spacing	Retrieval practice	− 4.785	2413	< 0.001	− 0.083	0.017
Low Elaboration	Low-Highlighting	20.956	2413	< 0.001	0.262	0.013
Low Elaboration	Low-Rereading	16.649	2413	< 0.001	0.147	0.009
Low Elaboration	Low-Rote learning	− 23.503	2413	< 0.001	− 0.359	0.015
Low Elaboration	High Elaboration	− 22.624	2413	< 0.001	− 0.288	0.013
Low Elaboration	High-M. comprehension	− 39.322	2413	< 0.001	− 0.586	0.015
Low Elaboration	High-Associating	− 5.722	2413	< 0.001	− 0.108	0.019
Low Elaboration	High-Processing	− 5.269	2413	< 0.001	− 0.076	0.014
Low Elaboration	Retrieval practice	1.520	2413	0.129	0.021	0.014
Low-Highlighting	Low-Rereading	− 6.717	2413	< 0.001	− 0.115	0.017
Low-Highlighting	Low-Rote learning	− 25.507	2413	< 0.001	− 0.621	0.024
Low-Highlighting	High Elaboration	− 29.566	2413	< 0.001	− 0.550	0.019
Low-Highlighting	High-M. comprehension	− 39.436	2413	< 0.001	− 0.848	0.022
Low-Highlighting	High-Associating	− 15.844	2413	< 0.001	− 0.370	0.023
Low-Highlighting	High-Processing	− 18.888	2413	< 0.001	− 0.338	0.018
Low-Highlighting	Retrieval practice	− 12.072	2413	< 0.001	− 0.241	0.020
Low-Rereading	Low-Rote learning	− 23.682	2413	< 0.001	− 0.506	0.021
Low-Rereading	High Elaboration	− 28.664	2413	< 0.001	− 0.435	0.015
Low-Rereading	High-M. comprehension	− 44.260	2413	< 0.001	− 0.733	0.017
Low-Rereading	High-Associating	− 12.628	2413	< 0.001	− 0.255	0.020
Low-Rereading	High-Processing	− 12.679	2413	< 0.001	− 0.223	0.018
Low-Rereading	Retrieval practice	− 7.058	2413	< 0.001	− 0.125	0.018
Low-Rote learning	High Elaboration	3.479	2413	0.028	0.070	0.020
Low-Rote learning	High-M. comprehension	− 10.810	2413	< 0.001	− 0.227	0.021
Low-Rote learning	High-Associating	10.158	2413	< 0.001	0.251	0.025
Low-Rote learning	High-Processing	12.874	2413	< 0.001	0.283	0.022
Low-Rote learning	Retrieval practice	20.248	2413	< 0.001	0.380	0.019
High Elaboration	High-M. comprehension	− 35.261	2413	< 0.001	− 0.297	0.008
High Elaboration	High-Associating	15.608	2413	< 0.001	0.180	0.012
High Elaboration	High-Processing	23.911	2413	< 0.001	0.213	0.009
High Elaboration	Retrieval practice	29.674	2413	< 0.001	0.310	0.010
High-M. comprehension	High-Associating	26.794	2413	< 0.001	0.478	0.018
High-M. comprehension	High-Processing	33.607	2413	< 0.001	0.510	0.015
High-M. comprehension	Retrieval practice	44.649	2413	< 0.001	0.607	0.014
High-Associating	High-Processing	1.903	2413	1	0.032	0.017
High-Associating	Retrieval practice	7.519	2413	< 0.001	0.130	0.017
High-Processing	Retrieval practice	8.252	2413	< 0.001	0.097	0.012

Student's t-test
