# Supplementary material for: Which learning techniques supported by cognitive research do students use at secondary school? Prevalence and associations with students’ beliefs and achievement
Source: Cogn Res Princ Implic. 2024 Jul 6;9:44. doi: 10.1186/s41235-024-00567-5 (PMC11227488; doi:10.1186/s41235-024-00567-5)
Supplement: Supplementary file 1 — Additional file 1. Distribution of scores: prevalence of use of the study techniques and prevalence of beliefs towards learning. [file 41235_2024_567_MOESM1_ESM.docx]

**Figure 2 (Supplementary Material)**

Distribution of Scores: Prevalence of Use of the Study Techniques


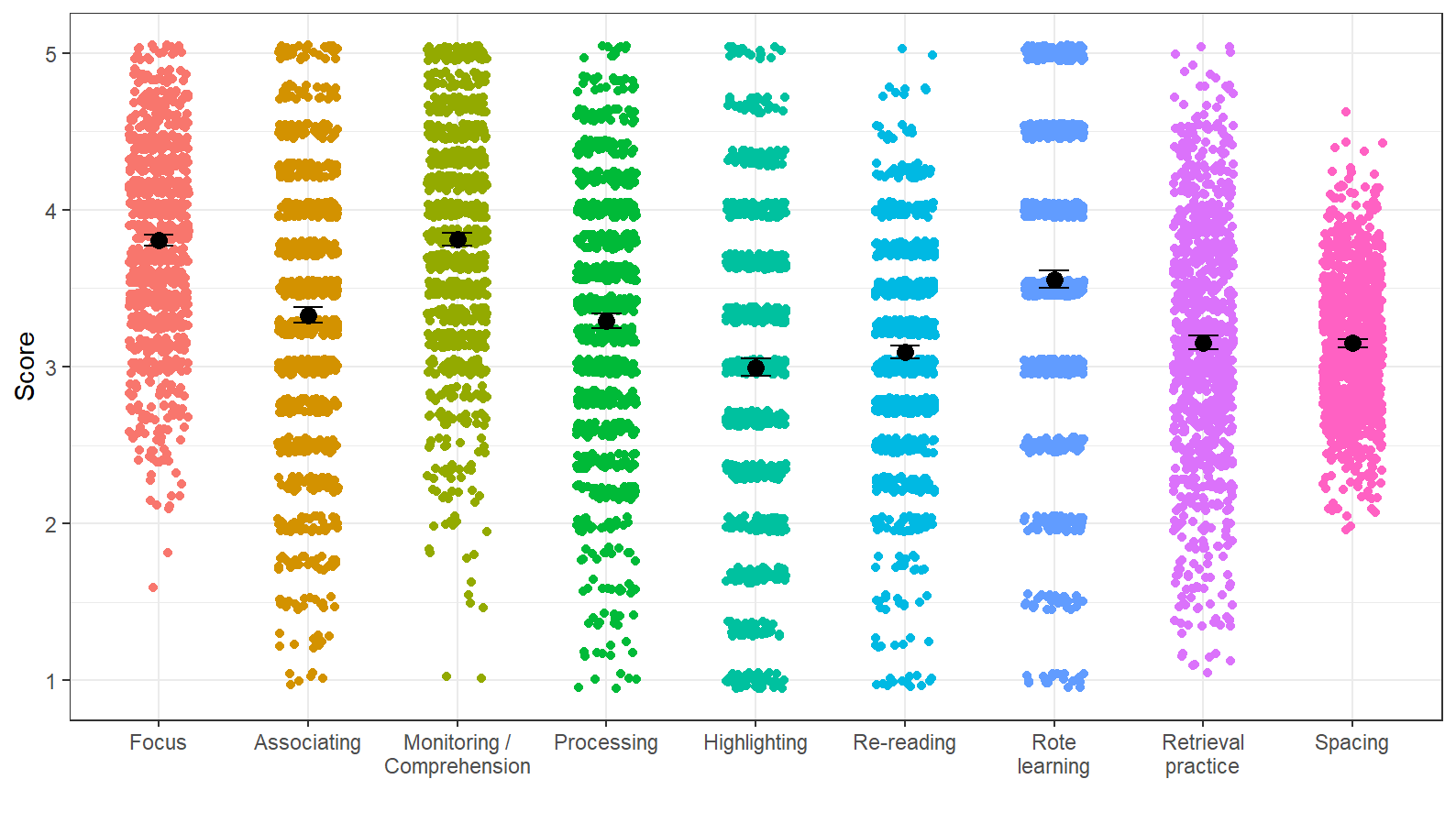


*Note.* Black dots represent the mean scores. Error bars depict 95% confidence intervals for the mean. Jitter has been added to data points to avoid overplotting.

**Figure 3 (Supplementary Material)**

Distribution of Scores: Prevalence of Beliefs Towards Learning

**
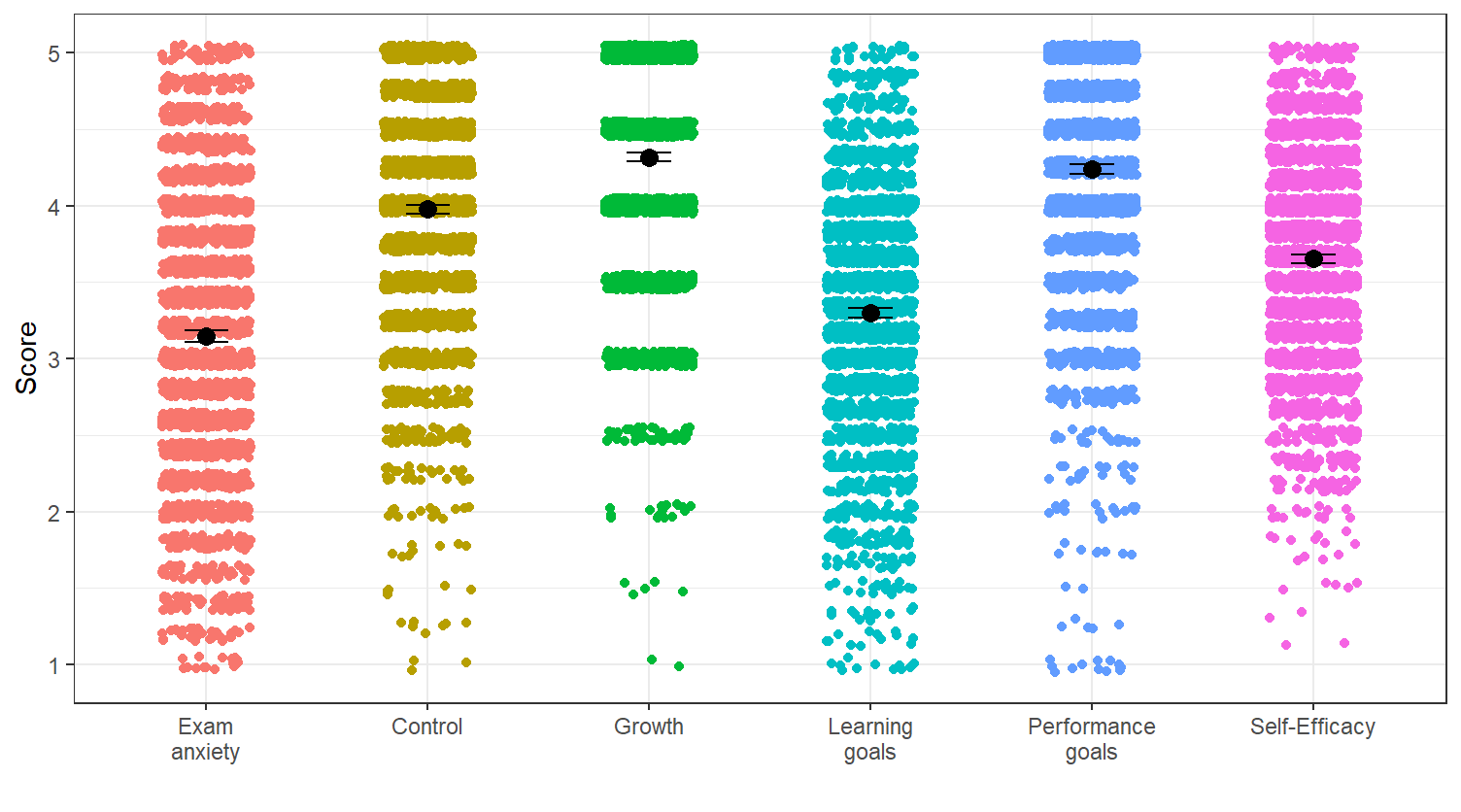
**

*Note.* Black dots represent the mean scores. Error bars depict 95% confidence intervals for the mean. Jitter has been added to data points to avoid overplotting.
